# Highlights from the Inaugural International Cancer Microbiome Consortium Meeting (ICMC), 5–6 September 2017, London, UK

**DOI:** 10.3332/ecancer.2017.791

**Published:** 2017-12-20

**Authors:** Alasdair J Scott, Claire A Merrifield, James L Alexander, Julian R Marchesi, James M Kinross

**Affiliations:** Department of Surgery and Cancer, Imperial College London, Kensington, London SW7 2AZ, UK

**Keywords:** microbiome, microbiota, cancer, gut, consortium

## Abstract

The International Cancer Microbiome Consortium (ICMC) is a recently launched collaborative between academics and academic-clinicians that aims to promote microbiome research within the field of oncology, establish expert consensus and deliver education for academics and clinicians. The inaugural two-day meeting was held at the Royal Society of Medicine (RSM), London, UK, 5–6 September 2017. Microbiome and cancer experts from around the world first delivered a series of talks during an educational day and then sat for a day of roundtable discussion to debate key topics in microbiome-cancer research.

Talks delivered during the educational day covered a broad range of microbiome-related topics. The potential role of the microbiome in the pathogenesis of colorectal cancer was discussed and debated in detail with experts highlighting the latest data in animal models and humans and addressing the question of causation versus association. The impact of the microbiota on other cancers—such as lung and urogenital tract—was also discussed. The microbiome represents a novel target for therapeutic manipulation in cancer and a number of talks explored how this might be realised through diet, faecal microbiota transplant and chemotherapeutics.

On the second day, experts debated pre-agreed topics with the aim of producing a consensus statement with a focus on the current state of our knowledge and key gaps for further development. The panel debated the notion of a ‘healthy’ microbiome and, in turn, the concept of dysbiosis in cancer. The mechanisms of microbiota-induced carcinogenesis were discussed in detail and our current conceptual models were assessed. Experts also considered co-factors in microbiome-induced carcinogenesis to conclude that the tripartite ‘interactome’ between genetically vulnerable host, environment and the microbiome is central to our current understanding. To conclude, the roundtable discussed how the microbiome may be exploited for therapeutic benefit in cancer and the safety implications of performing such research in oncology patients.

The ICMC was formed in 2017 with the aims of establishing an international expert consensus on the interaction between the human microbiome and cancer and its exploitation for therapeutic benefit. To that end, the inaugural ICMC meeting was held at the Royal College of Medicine, London, UK on 5–6 September 2017. The two-day meeting comprised a one-day educational event with lectures delivered by international experts to a broad-ranging, clinical and non-clinical audience highlighting cutting-edge research on the role of the microbiome in benign and malignant disease. On the second day, experts met for a roundtable discussion to develop a consensus statement regarding the role of the microbiome in carcinogenesis, with a focus on the current state of our knowledge and key gaps for further development.

## Day one—‘The microbiome in cancer and beyond’ educational event

The educational event was run in conjunction with the Trainees Section of the RSM and saw 16 academics and academic-clinicians from Europe, the Americas and Asia deliver a wide range of talks on the clinically relevant aspects of microbiome research. While the focus was on malignant disease, talks covered a variety of disciplines, including gynaecology, respiratory medicine, surgery, gastroenterology and pharmaceuticals. The day was attended by approximately 100 delegates and the broad appeal of microbiome research was reflected in the varied backgrounds of the attendees: clinicians, basic scientists, medical students, PhD students and veterinarians. It is unusual to have speakers of such diverse international origins at a relatively small meeting and it afforded an intimate atmosphere in combination with talks by world-leading experts.

The first session focussed on the role of the gut microbiome as an aetiological agent in colorectal cancer, almost certainly the most well-developed area of microbiome-oncology research. Professor Julian Marchesi (Professor of Digestive Health at Imperial College London, UK) and Dr Harpreet Wasan [Gastrointestinal (GI) lead for Medical Oncology at Imperial College Healthcare NHS Trust, UK] set the scene with introductory talks on the technical aspects of performing microbiome research—defining key terms (Box 1) and delineating the benefits and drawbacks of metagenomics and 16S rRNA gene-based metataxonomics over traditional culture-based techniques—and the current paradigms of colorectal carcinogenesis. Professor Christian Jobin (Professor of Medicine, University of Florida College of Medicine, USA) built on this by highlighting mechanisms by which commensal GI bacteria, the so-called ‘enemy within’, may promote carcinogenesis, including elegant work in gnotobiotic mice demonstrating how the *Escherichia coli*-derived colibactin virulence factor induces host DNA damage and drives tumourigenesis [1, 2]. Professor Rex Gaskins (Professor of Immunobiology, University of Illinois at Urbana-Champaign, USA) reinforced this notion in an illuminating lecture describing how hydrogen sulphide, produced by commensal sulphate-reducing bacteria from the metabolism of dietary sulphated amino acids, has genotoxic, pro-inflammatory and pro-carcinogenic effects [3]. There followed a lively debate session, chaired by Professor Julian Teare (Professor of Gastroenterology, Imperial College London, UK), titled, ‘*The microbiome and cancer: driver or passenger?’.* Dr David Hughes (Honorary Lecturer, Royal College of Surgeons in Ireland) and Professor Jun Yu (Professor in the Department of Medicine and Therapeutics, Chinese University of Hong Kong) used data from a number of human studies [4–6] to present strong arguments in favour of the gut microbiome as a driver of human colorectal cancer. Playing devil’s advocate were Professors Robert Brown (Chair in Translational Oncology and Head of Division of Cancer, Imperial College London, UK) and Luis Mur (Professor and Director of Research of the Biology and Health Theme, Aberystwyth University, Wales, UK) who cautioned that, although there is substantial evidence *associating* the microbiome with various malignancies [7], direct evidence of *causation* in humans remains to be proven and will require carefully designed longitudinal cohort studies. Although the audience (and possibly panel members) were perhaps somewhat biased, there was a general consensus that the human microbiome almost certainly has a role to play in carcinogenesis, though its relative contribution compared to host genetics and other environmental factors is still to be quantified.

**Figure 1.**
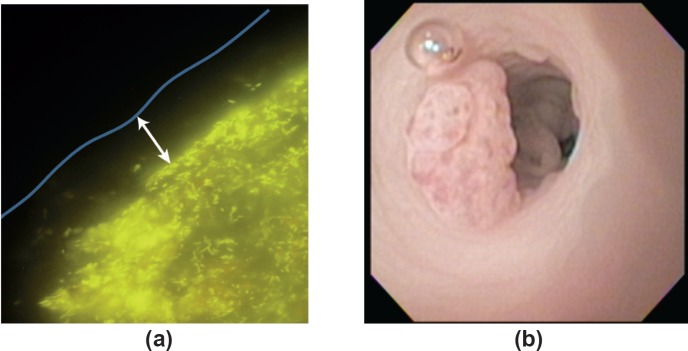
(a) Anatomy of the faecal cylinder. Hybridisation with Bac303 (Bacteroides) Cy3 (yellow fluorescence) probe. 10–100 μm bacteria-free mucus layer (white arrow) covers the faecal cylinder. This mucus limits direct contact between the bacteria and the colonic mucosa. Figure courtesy of Dr Alexander Swidsinski. (b) Colonoscopy in a mouse demonstrating colorectal tumour. Figure courtesy of Professor Daniel Rosenberg.

The first afternoon session saw an eclectic mix of talks showcasing microbiome research in various disease states. Dr Alexander Swidsinksi (Department of Medicine, Charité Universitätsmedizin Berlin, Germany) delivered a thought-provoking talk, supplemented with stunning fluorescence *in situ* hybridisation images (Figure 1a), on the functional anatomy of the human colon as a bioreactor and the key role of the gut mucus layer in providing physical separation between luminal microorganisms and colonic mucosa [8]. Professor Daniel Rosenberg (Chair in Cancer Biology and Professor of Medicine, University of Connecticut Health Centre, USA) also presented some fantastic pictures (Figure 1b) taken from colonoscopies performed in mice in his lecture describing how nutritive components of walnuts can promote the formation of a microbiome with antitumour properties in mice [9]. Moving away from the gut microbiome, Professor Hans Verstraelen (Professor of Urogynaecology, Ghent University Hospital, Belgium) described the vaginal microbiome and its potential role in cervical cancer [10], while Professor Jeremy Burton (Miriam Burnett Chair in Urological Sciences, Lawson Health Research Institute, Canada) presented his novel work on the microbiome of the urinary tract (which was thought to be sterile) and early suggestions of a role in urologic malignancies [11]. Professor Burton also highlighted the interesting link between the intestinal microbiome and urologic disease—metabolites and xenometabolites produced by the gut microbiome are often renally excreted and so can affect the urinary tract. Lastly, Professor Luis Mur described distinct microbiome changes in the sputum from patients with lung cancer and raised the possibility of bacterial biomarkers for lung cancer [7].

Box 1. **Key terminology in microbiome research (adapted from Marchesi *et al* 2015 [21]).**

**Bacterial cometabolites: **Biologically active substances, such as short chain fatty acids and secondary bile acids, produced by bacterial metabolism within the host, typically in the colon.

**Dysbiosis: **Departure from the healthy microbiome state. There is considerable inter-individual variability in human microbiomes and therefore dysbiosis is, at best, a relative term.

**Microbiome: **The functions or genes of a group of microbiota found in a given environment, e.g., the skin, mouth or colon. In man, the combination of these site-specific microbiomes comprises the human microbiome.

**Microbiota: **A term describing the total collection of microorganisms in a defined environment (e.g., the colon). May contain bacteria, archaea, fungi or viruses, although, to date, the majority of studies have focussed solely on bacteria.

**Metagenome: **The collection of all genomes and genes from members of the microbiota in a sample. Determined by shotgun sequencing of DNA and mapping of genes to a reference database. This allows potential characterisation of function of the microbiota.

**Metataxonomics: **A process performed using 16S rRNA gene sequencing to characterise all of the microbiota in a sample or group of samples. This allows construction of a metataxonomic tree, wherein the relative abundance of individual taxa can be quantified.

**Metabolome: **The complete set of biologically active elements or metabolites in a single strain or cell type.

**Metabonome: **The complete set of biologically active elements or metabolites in a system or fluid such as serum or faecal water.

**Pathobiont: **Members of the commensal microbiota that have the potential to cause disease.

**Pharmacomicrobiomics: **The bidirectional interaction between the host microbiota and drugs.

**Prebiotics: **Dietary components (e.g., non-digestible fibre) that potentiate the growth or activity of the microbiota.

**Figure 2.**
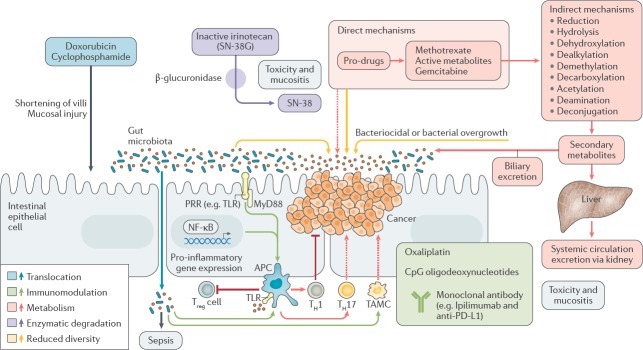
The TIMER framework summarises the mechanisms by which chemotherapeutics can interact with the gut microbiome. Translocation: cyclophosphamide can damage the mucosal barrier, permitting translocation of commensal bacteria. Immunomodulation: intestinal microbiota facilitate a plethora of chemotherapy-induced immune and inflammatory responses. For example, Lactobacillus mediates the accumulation of T-helper cell responses and bifidobacteria increase T-cell numbers in the tumour microenvironment in patients treated with anti-PD-L1 immunomodulator. Metabolism and enzymatic degradation: the microbiota harbour a large suite of metabolic processes (such as reduction, hydrolysis, dehydroxylation and dealkylation) which can modify pharmaceuticals to potentiate desirable effects, abrogate efficacy or liberate toxic compounds. For example, bacterial β-glucuronidases cleave the glucuronide from the inactive metabolite of irinotecan (SN-38G), releasing the active metabolite (SN-38) into the gut and causing diarrhoea. Reduced diversity: chemotherapy can modify the diversity of the intestinal microbiota through altered biliary excretion and secondary metabolism or associated antibiotic use and dietary modifications. The resulting expansion of pathobionts can lead to deleterious effects such as diarrhoea and colitis. PRR, pattern-recognition receptor; TAMC, tumour-associated myeloid cell; TLR, toll-like receptor; T_reg_ cell, regulatory T cell. Reproduced from Alexander *et al* [12] 2017 with kind permission.

The focus of the final session concerned manipulation of the microbiome for clinical benefit and was of particular interest to the clinical members of the audience. Mr James Kinross (Senior Lecturer and Consultant Colorectal Surgeon, Imperial College London, UK) delivered an insightful lecture on pharmacomicrobiomics—gut microbiota modulation of chemotherapeutic efficacy and toxicity. He proposed the TIMER mechanistic framework to explain microbiota-host-chemotherapeutic interactions—Translocation, Immunomodulation, Metabolism, Enzymatic degradation and Reduced diversity (Figure 2) [12]. Switching tack, Dr Benjamin Mullish (clinical research fellow, Imperial College London, UK) presented a thorough run-down of the evidence behind the use of faecal microbiota transplant in various disease states [13]. He raised several interesting issues regarding donor selection, safety, efficacy, legislation and mechanism of action that suggest that, while the therapy certainly has much potential, it is not yet the panacea the tabloid press might have one believe. Dietary manipulation of the gut microbiome may be an obvious proposition but high-quality studies in humans are notoriously difficult. The dietary swap study, presented by Professor Steve O’Keefe (Division of Gastroenterology, Hepatology and Nutrition, University of Pittsburgh, USA), was all the more remarkable therefore for being carried out in rural Africa and delivering convincing histologic, metabonomic and meta-taxonomic data showing that diet can rapidly shift the microbiota towards a more or less carcinogenic phenotype [14]. Finally, Professor John Alverdy (Sara and Harold Lincoln Thompson Professor of Surgery, University of Chicago, USA), challenged the widely held dogma that anastomotic leak after GI surgery is largely due to surgical technique. Rather, Professor Alverdy posited via a series of elegant investigations that collagenase-producing bacteria (such as *Enterococcus faecalis* and *Pseudomonas aeruginosa*) can cause leak independent of anastomotic technique [15, 16]—this will be music to the ears of self-castigating GI surgeons around the world.

In summary, the educational aspect of the ICMC meeting provided a detailed, yet broad overview on key, contemporary aspects of microbiome research with a focus on cancer. It was also a fantastic forum for debate and discussion between the audience and speakers, which frequently spilled out into the breaks between sessions. Interviews with many of the speakers are available on the *e*cancer website (http://ecancer.org/conference/1014-microbiome-in-cancer-and-beyond.php), and a selection of talks are available to watch in full on the RSM website (https://videos.rsm.ac.uk). The talks demonstrated that microbiome research is currently undergoing significant expansion to address new environmental niches and pathologies, making for an exciting, multidisciplinary science. The potential to increase our understanding of the pathogenesis of malignancy is considerable, as is the potential for clinical benefit. The ICMC was formed with exactly this in mind and convened for its inaugural roundtable discussion the following day.

Box 2. **Agenda for discussion by the roundtable.**

**Session One: Output from ICMC**

- As a consortium of experts with an interest in the microbiome, what should we aim to achieve and how might we collaborate constructively in this regard?
- When considering the ‘microbiome’ as an aetiological agent in cancer, can we create a useful framework that acknowledges carcinogenesis induced by pathogens—such as H. pylori and HPV—compared to pathobiont-associated cancer resulting from dysbiosis?

**Session Two: Molecular mechanisms of microbiome-induced carcinogenesis**

- Microorganisms may influence carcinogenesis in a variety of ways. For example, promoting inflammation, impairing immunity, genotoxicity and altered metabolism. What are the broad molecular mechanisms by which microorganisms may cause cancer—can we classify them?
- The ‘driver/passenger model’ and the ‘alpha-bug hypothesis’ are two conceptual models by which dysbiosis may cause colorectal cancer. Are these models generalisable to other cancers or do we need other models? Is there a unifying model to describe how dysbiosis may cause cancer in general?
- With respect to dysbiosis and carcinogenesis, have we moved beyond association to causation?

**Session Three: Co-factors in microbiome-induced carcinogenesis**

- Are there host genetic and/or epigenetic factors which influence the oncogenic potential of the human microbiome?
- How do environmental factors—such as diet, smoking, stress, exercise, drugs and alcohol amongst others—interact with the microbiome to promote carcinogenesis?

**Session Four: Safe exploitation of the microbiome for therapeutic benefit**

- How might the microbiome be exploited for therapeutic benefit in cancer prevention, diagnosis, prognostication and treatment?
- How can we research microbiome manipulation in oncology patients? Does this have patient safety implications?

## Day two—expert roundtable discussion on the role of the human microbiome in carcinogenesis

In the roundtable discussion, contributors addressed pre-agreed questions regarding the role of the human microbiome in carcinogenesis (Box 2). Selected experts delivered short talks to address a specific question and stimulate discussion. Other designated members of the roundtable had also considered the question in advance and provided further views before the question was opened to the roundtable for debate and formation of an expert consensus opinion.

Questions were divided into four sessions. Session one concerned the aims, objectives and output of the consortium. Mr Kinross led the discussion and defined the ICMC mission statement as, ‘the promotion of excellence in microbiome cancer research’. Principal functions of the consortium were agreed as forming and publishing expert consensus on key and contentious issues in microbiome-oncology research; providing education to engage with the scientific, clinical and lay communities; establishing collaboration with the formation of research networks and sharing of resources; lobbying and promoting cancer-microbiome research to governments, industry and patient groups; and raising funding through relationships with funding bodies and industry partners. One specific area of output the consortium was keen to deliver considered the conceptual framework we currently use to describe the microbiome as an aetiological agent in cancer. For instance, cancer may be caused by infection with so-called ‘pathogens’ (such as human papilloma virus (HPV) or *Helicobacter pylori*) or may be promoted by the community structure of the commensal microbiome (loosely termed dysbiosis, i.e., ‘the enemy within’). There followed an interesting debate as the roundtable, led by Professor Verstraelen, questioned the very nature of the ‘healthy’ human microbiome and, by extension, the definition of dysbiosis. After all, can we even define ‘health’ in an individual whose future phenotype is unknown and perhaps unknowable? Furthermore, the ‘gut’ microbiome is largely being profiled by studies on the colon and typically by faecal analysis. The microbiota of the rest of the GI tract and mucosal-associated microbes in particular is far less studied. Although opinion was divided on this fundamental issue, there was some agreement that the ‘healthy’ microbiome might best be described as a concept of ideals and dysbiosis as a departure from this ideal. We know that there is significant inter-individual variability with regard to the composition of the gut microbiome, and future studies may well need to consider dysbiosis as relative to the individual concerned rather than an absolute.

**Figure 3.**
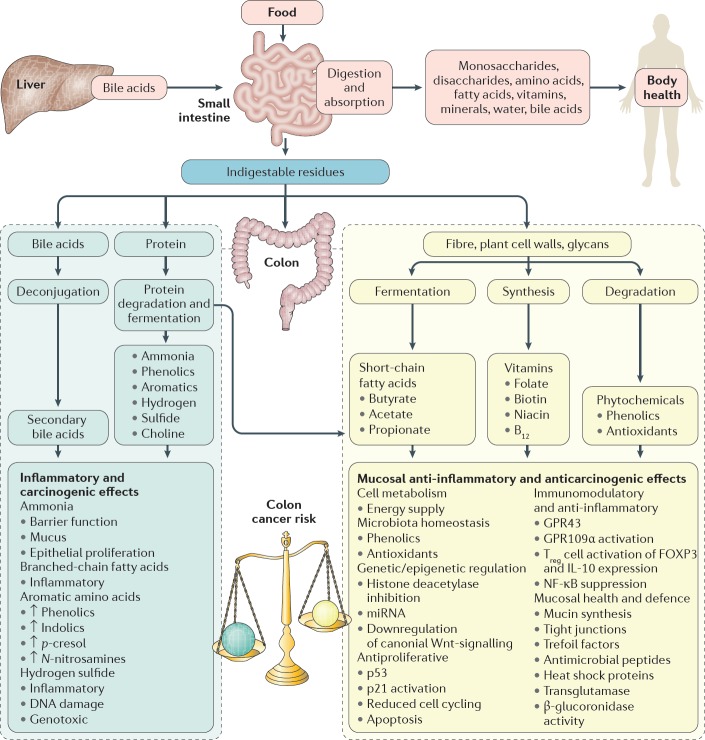
Interaction between diet and the microbiota results in the production of co-metabolites which can modify colon cancer risk. Saccharolytic fermentation of carbohydrates in fibre generates short-chain fatty acids, such as butyrate, which has anti-inflammatory and anti-neoplastic properties as indicated. On the other hand, a high fat/high animal protein diet promotes the formation of secondary bile acids and toxic nitrogenous and sulphur metabolites, which are pro-neoplastic. Reproduced from O’Keefe *et al* [17] 2016 with kind permission.

The second session focussed on the molecular mechanisms of microbiome-induced carcinogenesis. In the initial discussion, Professors O’Keefe and Gaskins highlighted the role of bacterial cometabolites, produced by dietary fermentation, as drivers of gut mucosal health and disease. The short chain fatty acid butyrate was cited as a prime example as it is the principal energy source for colonocytes and has anti-inflammatory, immunomodulatory and anti-neoplastic properties [17]. Hydrogen sulphide was also noted for its pro-inflammatory and genotoxic effects (Figure 3) [3]. Notably, the effects of these metabolites need not be confined to the GI tract and could be implicated in tumourigenesis at remote sites such as the urogenital tract. Inflammation, subversion of the immune system and toxin-induced genotoxicity were other proposed mechanisms. Professor Marchesi instigated discussion regarding how we might incorporate these mechanisms into a conceptual model of microbiome-induced carcinogenesis. Existing models of colorectal carcinogenesis propose that ‘keystone-pathogens’, ‘alpha-bugs’ or ‘drivers’ promote DNA damage and genomic instability by the aforementioned mechanisms, while subsequent tumour-associated microenvironmental changes may favour colonisation by ‘passenger’ species which may be innocent bystanders or further contribute to cancer progression [18]. Professor Yu pointed out that depletion of ‘protective’ species from the early tumour microenvironment may also be important. However, there was general consensus that these models probably over-emphasise the role of the microbiome at the expense of the host and we might be better served by considering a tripartite interactome of vulnerable host, environment and microbiome. Professor Jobin concluded the session, summarising the evidence that the microbiome can cause cancer. There was overwhelming evidence for the role of specific pathogens, such as HPV, *H. pylori* and Hepatitis B virus, but the evidence for the role of the commensal microbiome in carcinogenesis was less substantive. Experimental work in cell lines and animal models certainly provide plausible mechanisms but current human data, although compelling, are generally associative. The roundtable consensus was that, while the currently available data are persuasive, large and international, longitudinal cohort studies are needed to be more confident of causation rather than association.

A recurrent theme throughout the roundtable discussion was the ‘vulnerable host’. This term encapsulated the notion that the host microbiome alone is not able induce carcinogenesis, but rather that there must be host vulnerabilities that can render host–microbiome interactions carcinogenic. In the penultimate session, Dr Hughes explored the two-way interaction between the host genome and microbiome. On the one hand, a number of studies have demonstrated correlations between a host allele and a specific microbial phenotype, notably involving genes with immunoregulatory functions such as pattern recognition receptors and their adapter proteins [19]. On the other hand, the microbiota can induce host epigenetic changes (e.g., via histone deacetylase expression and modulation of host micro-RNA expression) and therefore modify host gene expression. Environmental factors too can render the host vulnerable to their microbiome. Swidsinski *et al* [8] explained his core hypothesis that colorectal carcinogenesis principally results from a direct contact between the luminal microbiota and the mucosa due to loss of the protective mucus barrier layer. He postulated that disruption of the mucus layer (e.g., by smoking or the emulsifying agent carboxymethylcellulose, which is found in many Western foodstuffs) may be a common final pathway by which many environmental agents interact with the microbiome to induce inflammation and cancer. While acknowledging the importance of the mucus layer, Professor O’Keefe proposed that the effect of the Western diet on colon cancer risk was more likely explained by the bacterial co-metabolism—low fibre intake compromising short-chain fatty acid (e.g., butyrate) production and high animal protein/fat intake resulting in the formation of toxic nitrogenous and sulphur metabolites. Professor O’Keefe pointed out that metabolites would generally penetrate the mucus layer and exert effects on the underlying mucosa.

The roundtable finished with a session, chaired by Professor David Cunningham (Consultant oncologist and Director of Clinical Research at The Royal Marsden, London, UK), exploring how the microbiome might be safely exploited for therapeutic benefit in the prevention, diagnosis and treatment of cancer. Professor Burton elucidated the broad tools at our disposal to manipulate the microbiome, including diet and supplements, surgery (e.g., gastric bypass), faecal transplant, antibiotics, bacteriophages and bacteriocins. With respect to diagnosis and prognosis, there are efforts to use faecal 16S rRNA gene sequencing as a biomarker for colorectal cancer [6] and the microbiome profile can be correlated with the T stage (Alexander *et al* [12], unpublished data). Dr Jessica Younes (Science Liaison, Winclove Probiotics, The Netherlands) gave an industry perspective on the safety concerns of manipulating the microbiome in oncology patients. Pro- and prebiotics certainly hold promise as therapeutic agents in this patient cohort, particularly with regard to the treatment of radiation-/chemotherapy-induced enteritis/colitis. However, these patients are especially vulnerable given their immunocompromised state and there is concern that the use of pro- or prebiotics may represent a hazard, as was seen with the use of probiotics in patients with severe acute pancreatitis (PROPATRIA trial [20]). Despite these concerns, the consortium felt that they could be safely trialled with appropriate caveats; probiotic strains should be extensively characterised *in vitro;* effects on the microbiome and metabonome should be explored in healthy, immunocompetent hosts; and these agents should be treated as drugs and corresponding good clinical practice followed.

This inaugural ICMC meeting represented the first attempt to bring together scientists and academic clinicians from different fields and different countries whose common interest is cancer and the microbiome. We delivered our twin aims of highlighting the importance of the cancer microbiome in an educational forum and debating key questions in the field. The consortium now aims to publish an expert consensus statement on the role of the microbiome in carcinogenesis and to develop further strategies for achieving our longer term goals with respect to delivery of education, forming research collaboratives and leveraging funding.

## Conflicts of Interest

The authors declare no conflicts of interest which could inappropriately influence the presented work.

## Funding

The meeting was sponsored by Winclove Probiotics, Protexin, Host Therabiomics and Yakult.
